# Análisis del encuadramiento periodístico en tiempos de pandemia de covid-19 en los principales diarios de Colombia: *El Tiempo* y *El Espectador*

**DOI:** 10.18294/sc.2024.4593

**Published:** 2024-03-15

**Authors:** Paola Consuelo Ladino Marín, Rodolfo Prada Penagos

**Affiliations:** 1 Doctora en Educación. Docente Investigadora, Facultad de Arte, Comunicación y Cultura, Universitaria Agustiniana, Bogotá, Colombia. paoladino2@hotmail.com Universitaria Agustiniana Facultad de Arte, Comunicación y Cultura Universitaria Agustiniana Bogotá Colombia paoladino2@hotmail.com; 2 Doctor en Ciencia de la Comunicación. Docente Investigador, Facultad de Arte, Comunicación y Cultura, Universitaria Agustiniana, Bogotá, Colombia. rodolfo.prada@uniagustiniana.edu.co Universitaria Agustiniana , Facultad de Arte, Comunicación y Cultura Universitaria Agustiniana Bogotá Colombia rodolfo.prada@uniagustiniana.edu.co

**Keywords:** Noticias, Medios de Comunicación de Masas, Covid-19, Colombia, News, Mass Media, Covid-19, Colombia

## Abstract

Se presenta un análisis de contenido de los titulares publicados en las portadas de los dos diarios de mayor tradición en Colombia durante la emergencia sanitaria por covid-19. El objetivo fue analizar el encuadramiento o framing de los periódicos El Tiempo y El Espectador durante el cubrimiento periodístico del primer periodo de la pandemia. Se tomaron como criterios de análisis el tono informativo, los abordajes temáticos, el tipo de estructura gramatical en los titulares, las fuentes de información, la cobertura y la perspectiva mediática. En relación con los marcos de interpretación, los medios privilegiaron un enfoque negativo de la crisis y del impacto estructural que la pandemia desencadenó en el país, sobre todo desde un contexto marcado por la incertidumbre y la tensión. Los principales temas se asociaron a lo económico y social. Los términos que tuvieron mayor visibilidad en los titulares de dicho periodo se asociaron con pandemia, covid-19 y virus, conceptos que transversalmente mantuvieron una marcada presencia en la agenda mediática de los medios objeto de estudio.

## Abstract (en)

This study presents a content analysis of headlines featured on the front pages of Colombia’s two most prominent newspapers during the COVID-19 health emergency. The objective was to examine the framing strategies employed by El Tiempo and El Espectador during their journalistic coverage of the initial phase of the pandemic. Criteria for analysis included informational tone, thematic approaches, grammatical structures in the headlines, information sources, coverage scope, and media perspective. Regarding interpretative frameworks, both newspapers predominantly adopted a negative approach towards the crisis and the structural impact the pandemic had on the country, especially within a context marked by uncertainty and tension. Primary themes were associated with economic and social aspects. The most prominent terms in the headlines during this period were linked to the pandemic, Covid-19, and the virus, concepts that consistently maintained a significant presence in the media agenda of the studied outlets.

## INTRODUCCIÓN

Desde comienzos de la década de 1970, cuando McCombs y Shaw^1^ formularon la noción de “*agenda setting*” y la teoría de los efectos de los marcos de interpretación que aplican los medios de prensa al definir sus agendas, demostraron que la información llega a las audiencias con sesgos que responden a los intereses editoriales e ideológicos de los emisores de los mensajes.

Frente a acontecimientos de relevante importancia, como los que se enmarcaron en la emergencia sanitaria suscitada por la aparición del covid-19, los medios aplicaron diversos enfoques que determinaron los contenidos periodísticos para narrar la pandemia. En este sentido, el interés de este estudio radicó en determinar de qué manera la prensa colombiana construyó la narrativa que las audiencias, afectadas directamente por lo sucedido, recibieron sobre lo que acontecía en el entorno local y global. 

Esta investigación partió del presupuesto de que los dos diarios de difusión de noticias más importantes de Colombia, con cobertura nacional (*El Espectador* y *El Tiempo*), produjeron y divulgaron sus contenidos a partir de prácticas de selección y encuadramiento, mediante las cuales construyeron ciertas representaciones de los aconteceres derivados de la pandemia.

Investigadores como Robert Entman^2^ y James Tankard^3^, que ampliaron los estudios de *agenda setting*, corroboraron los primeros hallazgos y ampliaron el espectro de las investigaciones en torno a los marcos de interpretación de los acontecimientos noticiosos. En esta dirección, años más tarde emergieron los estudios basados en la teoría del *fleming* que, según Natalia Aruguete^4^, han permitido el abordaje científico de las diferentes instancias vinculadas al proceso informativo, esto es: los medios y periodistas, los mensajes y las audiencias.

En este orden de ideas, el presente estudio se apoyó en la teoría del *framing* para indagar acerca de los enfoques, los marcos de interpretación y las miradas particulares que los diarios en referencia emplearon a la hora de presentar a sus audiencias los acontecimientos derivados de la pandemia.

De acuerdo con Andreu Casero-Ripollés^5^, la pandemia por el virus de covid-19 fue un suceso disruptivo que impactó directamente al periodismo. De hecho, la aparición del coronavirus se convirtió en un mega acontecimiento que tuvo impactantes consecuencias en distintas esferas socioeconómicas del mundo entero. Slavoj Žižek^6^, al reflexionar sobre la crisis, señaló que el coronavirus no solo desató la epidemia biológica, sino que activó otras de carácter ideológico que estaban latentes en la sociedad, como las teorías de la conspiración, la xenofobia y las noticias falsas.

Se trató de una crisis sanitaria global sin precedentes, que se originó por la enfermedad SARS-coV-2 y que, según la Organización Mundial de la Salud (OMS)^7^, tuvo su origen en China, donde por primera vez la Comisión de Salud Municipal de Wuhan en Hubei reportó, en diciembre de 2019, el primer caso de infección. De allí que millones de personas hayan perdido la vida y muchas otras padecieran las secuelas de un virus cuyos efectos resintieron sistémicamente la economía y las dimensiones sociales y políticas de todo el planeta, y que, en Colombia, evidentemente dejó un saldo importante de contagios y decesos. Al 22 de abril de 2023, el reporte oficial entregado por el Ministerio de Salud de Colombia daba cuenta de 6.364.636 de casos confirmados de contagio, 142.713 muertes y 6.187.047 recuperados^8^.

Partiendo de esta coyuntura, se consideró relevante analizar cómo los medios periodísticos, para el caso de Colombia, en particular los dos principales diarios -*El Tiempo*, como el periódico de circulación nacional más leído y, *El Espectador*, como el más antiguo de Colombia, con 135 años de circulación- informaban sobre esta situación que, en enero de 2020, reportó el primer caso de contagio, de acuerdo con el Ministerio de Salud^9^.

De modo que esta investigación se presenta como un aporte para el análisis del ejercicio periodístico escrito colombiano y como una forma de sistematizar y observar el manejo y encuadramiento (*framing*) que los medios tradicionales presentaron sobre los desarrollos de la pandemia, que resintió todas las estructuras sociales y ocupó lugares preponderantes en la agenda de todos los medios de comunicación. 

Según Pilar Giménez Armentia^10^, la teoría del *framing* se asocia con los distintos procesos informativos y elementos que intervienen cuando se publica un contenido periodístico, estableciendo secciones, palabras y hechos, específicamente en el mensaje; una forma de presentar la realidad al lector, televidente u oyente. 

Antes de hacer referencia a los temas, el tipo de cobertura, el tono, el lenguaje, la ubicación de las noticias y las fuentes que se consultaron para presentar este acontecimiento de orden mundial, es importante identificar conceptualmente la teoría del *framing*, ya que es la perspectiva teórica desde la cual se realizó este análisis de contenido. 

### Teoría del *framing*

La teoría del *framing* emerge en el área de la sociología interpretativa, y señala que “la interpretación que hacen los individuos de la realidad y de la vida cotidiana depende fundamentalmente de la interacción y de la definición de las situaciones”^11^. Según Teresa Sádaba^12^, esta teoría ha tomado fuerza en investigaciones de comunicación cuando el objeto de estudio son los medios, ya que permite analizar el comportamiento de estos y su establecimiento en la agenda noticiosa. Para Nadia Koziner^13^, la teoría se centra en los encuadres, entendidos como los atributos de los abordajes temáticos que van desde lo simple a lo complejo, y que hacen parte de diversos puntos de vista que predominan para organizar los datos acerca de las ideas que se generan sobre los objetos. 

Para Alberto Ardèvol-Abreu^11^, el *framing* o encuadre en el marco periodístico se puede aplicar a “cualquier texto comunicativo que requiere estructuras narrativas que organicen el discurso”. En el caso de los medios de comunicación, aplica porque los hechos noticiosos deben presentarse de forma sistematizada y fundamentada en convenciones narrativas propias del periodismo que ofrecen una explicación sobre el qué, el quién o el con qué intención determinado suceso se presenta a la audiencia.

### El ejercicio informativo en medio de la pandemia

Para Andreu Casero-Ripollés^5^, la práctica informativa tiene una importante repercusión no solo en el conocimiento que el ciudadano tiene de su entorno inmediato, sino también en términos democráticos, debido a la relación que tiene la información y la democracia. Es así como la noticia pasa a convertirse en un producto vital para la vida cívica, ya que el consumo de noticias es clave frente a la responsabilidad de una sociedad informada^14^. 

Declarada universalmente la pandemia, en los medios tradicionales y en las plataformas digitales se desataron cuantiosos flujos de información que sobresaturaron a las audiencias. Ante este estado de cosas, la OMS^15^ alertó sobre la denominada infodemia, entendida como una cantidad excesiva de información, a veces verídica, a veces no, que dificultó que las personas encontraran datos u orientaciones, a partir de fuentes confiables, cuando lo requerían. Este fenómeno suscitó la desinformación y la manipulación de datos con intenciones dudosas, lo cual se propagó en redes sociales, con la rapidez y el alcance con que avanzaba el virus^16^. 

Este aspecto causó honda preocupación, dada la facilidad y el acceso que las audiencias tuvieron a la información mediante los teléfonos celulares y otros dispositivos conectados a Internet, además de las redes sociales, donde se generó información exponencial, creando justamente otra epidemia: la informativa. Por esta razón, la OMS^15^ creó diversas formas para luchar contra la infodemia sobre el covid-19, manteniendo su web actualizada, divulgando datos científicos, apoyando la ciencia abierta y divulgando boletines informativos que recomendaron cómo no caer en la desinformación. Algunas de las pautas que se señalaron en ese momento propugnaban por corroborar las fuentes, sobre todo los hilos de WhatsApp; no compartir información sin validar; consultar datos de calidad y participar de forma responsable en las conversaciones sociales. 

Frente a dicho escenario, la ciudadanía reconoció la necesidad de conocer la verdad sobre lo que sucedía en medio de una sobreabundancia de datos. Tal como lo planteó Juan Benavides^17^, el tratamiento de datos se vio afectado, pues en un primer nivel se identificó un contenido cuantioso, en un segundo nivel se vio afectada la información verídica o aquellos datos fundamentales de acuerdo con lo transmitido y, en un tercer nivel, que tiene como finalidad el cruce de variables, se vio vulnerado el análisis, ya que no toda la información era transparente y veraz. 

En Colombia, la Fundación Gabriel García Márquez^18^ recomendó a los periodistas que cubrían la pandemia tener al Ministerio de Salud y Protección Social del país como fuente oficial, dado que contaba con líneas de atención y en su sitio web respondía preguntas frecuentes sobre la pandemia, los casos confirmados de contagio a nivel nacional y mundial, noticias, entre otras guías y protocolos. Por su parte, la Unesco^19^ destacó que el ejercicio periodístico era clave para suministrar información fehaciente en medio de tanta desinformación, y esto permitió combatir mitos y rumores. Vale la pena destacar que en la medida en que los medios comunicativos se fortalezcan, se puede ir despejando el peligro de la confusión y la discordia. Sin embargo, el tema se torna complejo cuando se reducen las fuentes válidas de información. 

Para la Fundación para la Libertad de Prensa^20^, el acceso al contenido público en Colombia durante la pandemia no se garantizó de manera plena por varias razones, entre ellas, los problemas de orden público, a raíz de las marchas sociales por la crisis económica en Colombia, que fueron la excusa para demorar el reporte de datos sobre los contagios y el limitado acceso a fuentes de información, pues el Estado señaló que las únicas fuentes con validación informativa eran el Instituto Nacional de Salud y el Ministerio de Salud, limitando el acceso a otras fuentes de información. 

Otras quejas que reportaron periodistas colombianos se relacionan con la falta de normas claras en el uso de plataformas digitales como Twitter, Facebook y WhatsApp, ya que en varias oportunidades en estos espacios utilizados por fuentes oficiales se bloqueaban comentarios, no se respondían preguntas y se excluían algunos medios por su línea editorial, tamaño y audiencia. De modo que esta práctica no permitió un flujo de información adecuado que respondiera a todas las necesidades informativas territoriales. Incluso, desde la Fundación para la Libertad de Prensa se hizo una solicitud a la Procuraduría General de la Nación de Colombia para establecer guías de uso, de manera de garantizar el acceso a información de calidad.

Rodolfo Prada *et al*.^21^ en su análisis multimodal de la representación de la pandemia en los titulares de los principales diarios de Colombia, señalan que las imágenes en los medios analizados se enfocaron en la situación de vulnerabilidad de sectores que aparecen representados “no solo como víctimas de la pandemia, sino como actores de una protesta que se reaviva por la crisis social y económica generada por el confinamiento sanitario”.

Para De Sousa Santos^22^, la situación planteada por la pandemia visibiliza grupos especialmente discriminados, que integran “el sur” olvidado, ignorado, perdido. Este autor explica que sur “no designa un espacio geográfico, sino un espacio-tiempo político, social y cultural”. Agrega que se trata de una “metáfora del sufrimiento humano injusto”, causado por la explotación capitalista y la discriminación racial y sexual. En este sentido, la pandemia se percibe como un contexto que cambió las dinámicas socioeconómicas a nivel mundial y en Colombia. Desde esta mirada, es interesante analizar cuáles son los contenidos en torno al covid-19 que en los dos más importantes diarios de Colombia se reconocieron en el primer periodo de la pandemia. 

Bajo estas consideraciones, se planteó la pregunta de investigación: ¿cuál fue el encuadramiento mediático del covid-19 en los principales medios de prensa escrita en Colombia?

## ASPECTOS METODOLÓGICOS

Se recopilaron los títulos de primera plana sobre la pandemia de covid-19 en los periódicos *El Espectador* y *El Tiempo*. La elección de los periódicos se basó en que *El Espectador* y *El Tiempo* son los medios de prensa con mayor tradición y reconocimiento en Colombia, ambos con más de un siglo de existencia y con amplia cobertura en el territorio nacional. *El Tiempo* publicó su primer ejemplar el 30 de enero de 1911, actualmente propiedad de la Casa Editorial el Tiempo S.A.; y *El Espectador* comenzó a circular el 22 de marzo de 1887, y actualmente es propiedad del Grupo Valorem.

La recuperación de los titulares se realizó de manera distinta para cada medio. En el caso de *El Tiempo*, se consultaron todas la primeras páginas impresas publicadas durante el periodo de análisis seleccionado. En cuanto a *El Espectador*, esta consulta se hizo mediante la cuenta oficial del diario en Instagram, en la cual se publican todas las portadas de la versión impresa del periódico. 

Se tuvo como criterio de selección que los titulares pertenecieran a notas periodísticas informativas, por lo que se excluyeron las notas de opinión. Esto obedece a que estas últimas recogen la mirada particular de su autor, que no necesariamente está ajustada a los criterios de encuadramiento del medio. Se analizaron todos lo titulares de los informes cuyo foco informativo se enmarcaba en los eventos relacionados directamente con la evolución de la pandemia. 

Como resultado de la fase inicial se estructuró un corpus que contempló 294 titulares de *El Tiempo* y 217 de *El Espectador*, para un total de 511 encabezados de primera plana analizados, comprendidos entre el 15 de marzo y el 15 de julio del 2020, periodo que abarca el comienzo y el primer pico de la pandemia en Colombia.

Se seleccionó dicho periodo por considerar que fue el lapso en el que los medios analizados enfrentaron un inesperado proceso de adaptación a la nueva coyuntura informativa, esta vez de largo aliento, en un país en el que las agendas cambian abruptamente al ritmo de las convulsiones políticas, económicas, sociales y de orden público. Se trató, además, de un acontecimiento global, que permeó todos los ámbitos de la vida social y que no tenía antecedentes en la historia del periodismo nacional. El 6 de marzo de 2020, cuando se presentó el primer caso de contagio en Colombia, se abrió un primer período que se cerró el 30 de julio del 2020, cuando el Ministerio de Salud reportó 276.055 infectados, 9.454 fallecidos y 142.777 recuperados, y se comprendió como la primera etapa de una emergencia sanitaria durante la cual el gobierno aplicó y puso a prueba las más inmediatas medidas de contención de la epidemia: cuarentena, uso de tapabocas, distanciamiento obligatorio y lavado permanente y minucioso de las manos. Las autoridades sanitarias señalaron varios picos de contagio, fijándose el 15 de julio como la fecha del primero de ellos. En una investigación realizada durante la pandemia, basada en un modelo matemático para predecir picos de contagio, Chaves Castro^23^ determinó que, tomando una ventana de tiempo de 60 días a partir del 12 de mayo, el primer pico de contagio se alcanzó en Colombia el día 15 de julio de 2020, fecha que se tomó para acotar el periodo de análisis para esta investigación.

Se optó por analizar los titulares en tanto son los elementos mayormente visibles de las noticias, contienen macro-proposiciones semánticas que resumen la macroestructura del texto^24^ y dan cuenta de los criterios de interpretación adoptados por el medio para enfocar el hecho noticioso. Esto último se relaciona directamente con la función apelativa del título que, según Beatriz Gallardo Paúls *et al*.^25^, consiste en estimular la curiosidad del lector. Aunque, por lo general, el título presenta un resumen de la noticia, también puede suceder que su intención sea la de destacar elementos que no necesariamente están explícitos en el texto, sino fuera de este. Esto se observa, por ejemplo, en *El Espectador*, en el que la carga semántica de los encabezados de portada se recupera en relación con el contexto. En este tipo de titulares, como lo señala Irene Vasilachis de Gialdano^26^, el enunciador acude a recursos como la metáfora o la ironía, que le exigen al lector ubicarse en el sistema cognitivo de referencia seleccionado por el periodista. Un argumento de fuerza adicional, y por el que muchos investigadores enfocan sus estudios en los titulares, es que gran parte de las personas que leen periódicos producen opiniones acerca de los hechos noticiosos solo a partir de la lectura que hacen de los titulares^27^.

Una vez estructurado el corpus, se procedió al registro de las unidades de análisis (los titulares) en una matriz bibliográfica elaborada en hojas de cálculo de Excel por cada uno de los medios seleccionados. Luego, se procedió a la cuantificación de las apariciones y frecuencias de los elementos en relación con las categorías, mediante el diseño de una matriz de análisis de contenido. En los procesos analíticos fue indispensable la aplicación de la herramienta de filtro de Excel.

Para el análisis del corpus se aplicó la técnica de análisis de contenido que, de acuerdo con Fernando López Noguero^28^, se vincula con un ejercicio analítico de fuentes documentales donde previamente se deben generar unos objetivos claros, concretos y precisos frente a lo que se busca reconocer en los documentos. Se trata, por demás, de un método que ha evolucionado en el campo de la investigación en comunicación, especialmente nutrido de corpus teóricos como el del *framing* y la teoría de *agenda setting*^29^.

Durante el proceso de investigación, los datos se filtraron y examinaron rigurosamente, con una reflexión permanente que contempló el entorno y el contexto referente. Para Flory Fernández^30^, el análisis de contenido de los documentos objeto de estudio deben contemplar los siguientes aspectos:

- *Importancia*: Documentos escritos con capacidad de convertirse en registros históricos. En el presente caso, las primeras planas de los dos principales diarios colombianos que cubrieron la pandemia, un hecho sin precedentes de la segunda década del siglo XXI, en todo el mundo.
- *Tipo de publicación*: Información periodística, traducida en titulares de primera plana de dos de los principales periódicos de circulación diaria en Colombia, con sus correspondientes plataformas de divulgación en la web.
- *Variedad de análisis de contenido* **:** a) Desde su aspecto formal (abordajes temáticos y tono informativo); b) por sus relaciones externas (fuentes periodísticas); desde sus características internas (tipo lenguaje, ubicación en el medio y cobertura).

El análisis de contenido se considera una herramienta objetivada por categorías o variables que responden al diseño de un estudio longitudinal con criterios de análisis definidos y explícitos que se utilizan para estudiar un documento. Para este estudio, se definieron las categorías incluidas en la Tabla 1. 

**Tabla 1:** Categorías principales y valores del análisis de los titulares de los periódicos *El Tiempo* y *El Espectador*. Colombia, 15 de marzo a 15 de julio de 2020.

Categoría principal	Categorías relacionadas
Tono	Positivo
	Neutro
	Negativo
Tema principal	Politico
	Económico
	Social
	Salud
	Educativo
	Religioso
	Deportivo
	Entretenimiento
Lenguaje	Denotativo
Connotativo	Connotativo
Fuentes (Tipología básica de fuentes)	Fuente Documental
	Fuente Testimonial
Cobertura	Nacional
	Internacional
Ubicación	Zona A - Zonas A y B - Zona B - Zonas B y D - Zona C - Zonas C y D - Zona D - Zonas A y D -Zonas A-B-C-D - Zonas A y C

Fuente: Elaboración propia a partir de Núñez-Gómez, Abuín-Vences y Sierra-Sánchez^31^.

Estos elementos llevaron a considerar que los criterios de encuadramiento aplicados por los medios a las noticias en esa primera etapa de la pandemia debieron de estar determinados por una fase de aprehensión cognitiva sobre la emergencia sanitaria, que puso a prueba la capacidad de reacción de los medios en un escenario de incertidumbre.

## RESULTADOS: ANÁLISIS Y DISCUSIÓN

**Figura 1:**
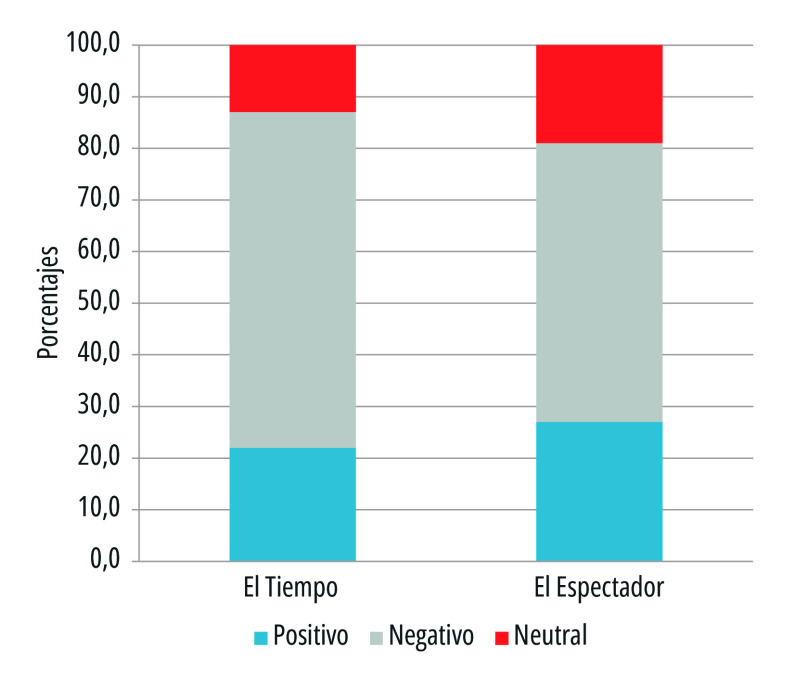
Tono de los titulares de primera plana de los periódicos *El Tiempo* y *El Espectador*. Colombia, 15 de marzo a 15 de julio de 2020

Un foco de análisis inicial fue el tono de los titulares periodísticos durante la crisis sanitaria por covid-19 en Colombia. Se encontró que ambos medios escritos con cobertura nacional tuvieron una inclinación hacia tono negativo, específicamente *El Tiempo*, con el 64% de informes, y *El Espectador*, con el 54%. Según Patrick Brown^32^, los medios proyectaron una visión más desastrosa que positiva, sobre todo en un tiempo en el que primó la incertidumbre (Figura 1).

Fuente: Elaboración propia

Titulares asociados a la crisis económica, el drama de la cuarentena, el confinamiento prolongado, las numerosas muertes, la emergencia sanitaria, los homicidios, los hurtos, la inconformidad educativa, el colapso en el sistema de salud, los abusos, la violencia intrafamiliar, la inflación y las cifras de contagio, entre otros hechos noticiosos, fueron los que hicieron un énfasis espacial en el encuadramiento negativo.

Este tono se intensificó en la fase inicial de la pandemia y en las fechas en las que se registraron picos de contagio, lo cual concuerda con lo planteado por Ahorsu *et al*.^33^, quienes han señalado que la aparición del covid-19 generó temores, preocupación y ansiedad en personas de todo el mundo y, en este sentido, los medios reflejaron dicha tendencia. 

El tono positivo fue, por consiguiente, menor: en *El Espectador* con el 27% de los registros, y en *El Tiempo* con el 22% (Figura 1). Esta tendencia hacia lo positivo está asociada al reconocimiento del trabajo del personal de la salud que ocupaba la primera línea de atención, a la esperanza de una pronta recuperación de los infectados, a la reactivación económica, al desarrollo tecnológico en el ámbito científico para contrarrestar el avance del virus, a las iniciativas culturales para disfrutar desde la virtualidad, a las medidas de prevención y a la ayuda humanitaria.

Estos enfoques se correspondieron con los momentos en que se registraron descensos en las cifras de contagio y el Gobierno tomaba medidas para tratar de contener el avance del virus. De esta manera, sobre la base de las nociones de la *agenda setting*, se evidenció una relación entre la agenda de los medios y la agenda pública, esta última determinada por los contenidos que los medios publicaban y el enfoque con el que lo hacían. En otras palabras, la respuesta de la gente a los temas que generan preocupación social en un contexto determinado puede estar condicionada por la influencia que ejercen los medios^34^.

El tono neutro sobre la pandemia en los titulares se registró en menor proporción: en el 13% de los encabezados de *El Tiempo* y en el 19% de los titulares de *El Espectador*. Se resaltan titulares de tipo informativo con intención educativa frente a las audiencias, en alusión a cómo se podían evitar contagios, la promoción del autocuidado y la pandemia en cifras.

En relación con los temas que abordaron los titulares de prensa de los periódicos analizados, el diario *El Espectador* tuvo un enfoque orientado más hacia lo social (31%), con títulos que destacaban aspectos de la familia en medio de la pandemia, la capacidad de superación y el desarrollo de diversas comunidades a nivel nacional. El segundo tema en relevancia fue el de salud (29%), con temas vinculados a las medidas sanitarias, necesidades del sector, talento humano de la salud, equipos y desarrollos tecnológicos en al área.

**Figura 2:**
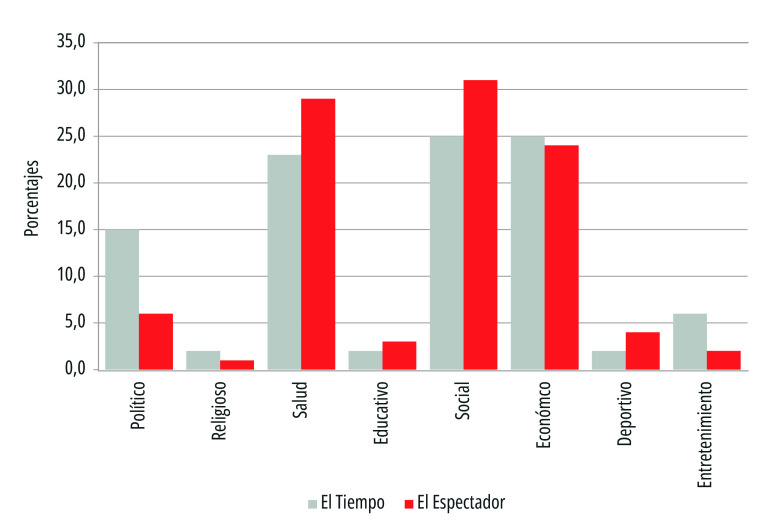
Temas centrales de los titulares de primera plana de los periódicos *El Tiempo* y *El Espectador*. Colombia, 15 de marzo a 15 de julio de 2020.

El tema económico emergió en un tercer lugar (24%) de frecuencia, con titulares acerca de recaudo, ajuste tributario y comportamiento de los subsectores. A su vez, el aspecto político se presentó en cuarto lugar (12%), en relación con la socialización de medidas que el Estado central tomaba frente a la crisis sanitaria. Los demás temas (deportivo, con 4%; entretenimiento, con 2%; y religioso, con 1%) vincularon abordajes culturales, alternativos, musicales, así como nuevas realidades para los deportistas y artistas, como el apoyo que la Iglesia Católica generó a las comunidades más vulnerables durante la pandemia (Figura 2).

Fuente: Elaboración propia.

Los medios seleccionan diversas fuentes y temas con el propósito de anticipar acciones que probablemente tomarán los actores políticos, es decir, que anticipan la agenda del gobierno. En este aspecto, la decisión de un medio de privilegiar ciertos temas sobre otros va en relación directa con los encuadres noticiosos sobre esos temas, es decir, que los análisis de los temas y el de los *frames* es de carácter complementario, no excluyente^35^.

En el estudio se observó que el *framing* del diario *El Tiempo* tuvo una mayor inclinación en aspectos económicos y sociales (25%), teniendo como referentes medidas desde el Gobierno central, como el comportamiento económico de diversos sectores que se vinculaban con la economía. En tercer y cuarto lugar se presentaron los temas de salud (23%) y política (15%), cuyos titulares giraban alrededor de medidas presidenciales, ministeriales y del Congreso. En relación con la salud, el enfoque se daba desde lo nacional e internacional con fuentes oficiales que hacían referencia especialmente a aspectos del desarrollo de la pandemia.

Otros abordajes se hicieron acerca de los temas relacionados con el entretenimiento (6%), la educación (2%), el deporte (2%) y la religión (2%), que generalmente visibilizaban aspectos positivos o esperanzadores, tanto a partir de fuentes institucionales privadas como públicas (Figura 2).

Otro aspecto relevante que se contempló en este análisis fue el tipo de estructura del titular desde lo denotativo y lo connotativo. La denotación se alinea a la realidad desde un sentido netamente objetivo, mientas que lo connotativo tiende a simbolizar o aportar emociones al lector. En este caso, durante el cubrimiento inicial de la pandemia, los medios privilegiaron titulares de carácter denotativo, soportados en fuentes oficiales, cifras y datos crudos.

**Figura 3.**
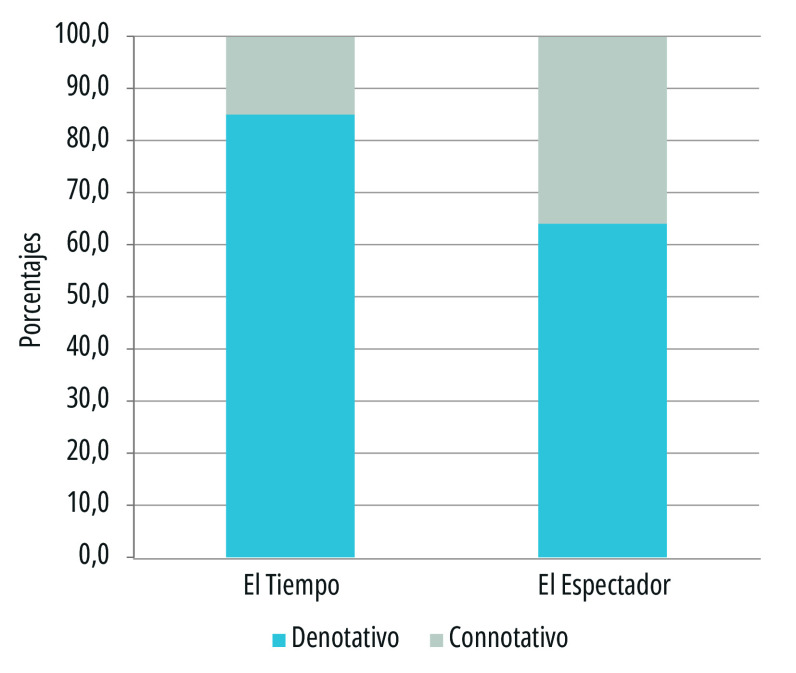
Tipos de estructura connotativa y denotativa en los titulares de primera plana de los periódicos *El Tiempo* y *El Espectador*. Colombia, 15 de marzo a 15 de julio de 2020.

En *El Tiempo*, el porcentaje de títulos en estructura denotativa fue del 85% en tanto que en *El Espectador* fue del 64%, basados preferencialmente en fuentes oficiales (Figura 3). En contraste, con el avance de la pandemia, se destacó un interés por motivar a las audiencias ante tanta información negativa, lo cual se asoció con titulares en estructuras connotativas que invitaban a la solidaridad, la resiliencia, la esperanza y la fe por un panorama más alentador.

Fuente: Elaboración propia.

Como se señaló antes, el título puede desempeñar una función apelativa en tanto supone un “reto a la comprensión”, como lo señalan Gallardo y Enguix^25^. En este sentido, y tratándose de textos que corresponden al género informativo, los titulares connotativos aportan una carga de subjetividad que es propia de los géneros de opinión, que dejan ver una intención por parte del enunciador que va más allá de la función meramente declarativa. *El Espectador* reflejó una mayor tendencia hacia este tipo de titulares, con el 36%, y en menor proporción *El Tiempo*, con el 15%. Este resultado se alineó con los temas, pues *El Espectador* tuvo mayor preferencia por temas sociales, en tanto que *El Tiempo*, por temas económicos. 

**Figura 4:**
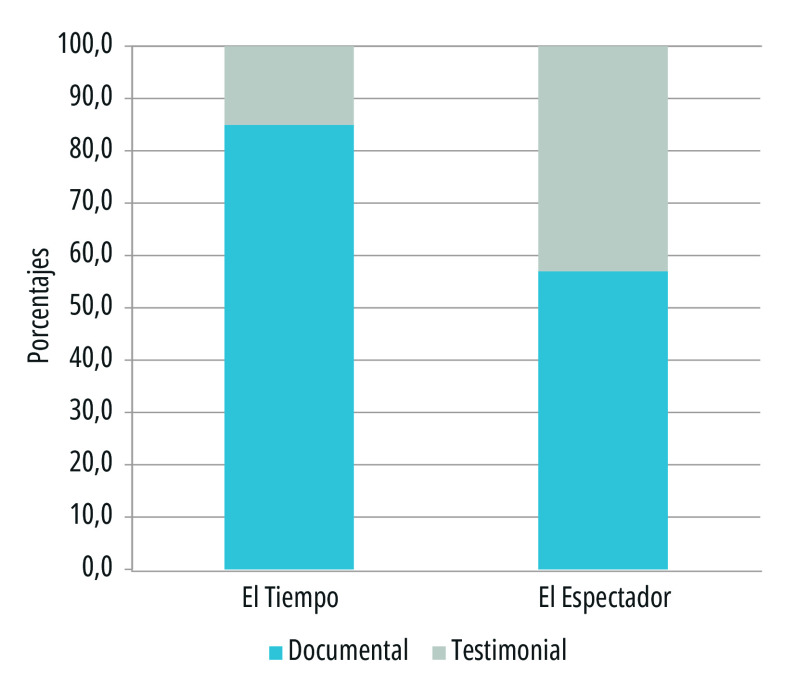
Tipos de fuentes testimoniales y documentales en los titulares de primera plana de los periódicos El Tiempo y El Espectador. Colombia, 15 de marzo a 15 de julio de 2020.

Dentro de los estudios sobre el *framing* en el ámbito de los contenidos de la prensa, el análisis de las fuentes de información cobra especial importancia, pues los medios, al seleccionar las fuentes que abordan, lo hacen a sabiendas del grado de autoridad que estas ostentan y la perspectiva desde la cual suministran la información solicitada. Las fuentes constituyen un componente esencial del proceso de producción de noticias. En las amplias tipologías elaboradas sobre las fuentes de acuerdo con su grado de cercanía al hecho, su procedencia institucional y su rol social, se identifican dos tipos de fuentes: las testimoniales y las documentales, considerándose que estas últimas aportan más confiabilidad a la noticia, mientras que las primeras, las testimoniales, le brindan actualidad y vivacidad al reporte periodístico. En este orden de ideas, se analizaron dos tipos de fuentes: las de orden testimonial y documental (Figura 4).

Fuente: Elaboración propia

En los medios analizados, *El Tiempo* sustentó el 85% de sus registros en fuentes documentales en tanto que *El Espectador* lo hizo en el 57% de los casos. En cuanto a las fuentes testimoniales, *El Tiempo* las utilizó en el 15% de los registros, mientras que *El Espectador* lo hizo en el 43%. Cabe señalar que el enfoque positivo de *El Espectador* se alineó más con temas de naturaleza social, que visibilizaban testimonios directos de quienes vivían la pandemia, vinculando fuentes expertas como científicos, médicos y figuras oficiales, pero también de representantes de comunidades, que le permitió al diario brindar la cara humana de la noticia (Figura 4). 

La cobertura periodística también se analizó desde el marco nacional e internacional, entendiendo que el virus del covid-19 fue un evento de naturaleza global y que, por ende, las agendas de noticias del planeta entero lo contemplaron como prioritario, además de que muchas de las cifras oficiales, como desarrollos científicos y tecnológicos, se daban desde escenarios intrafronterizos.

**Figura 5:**
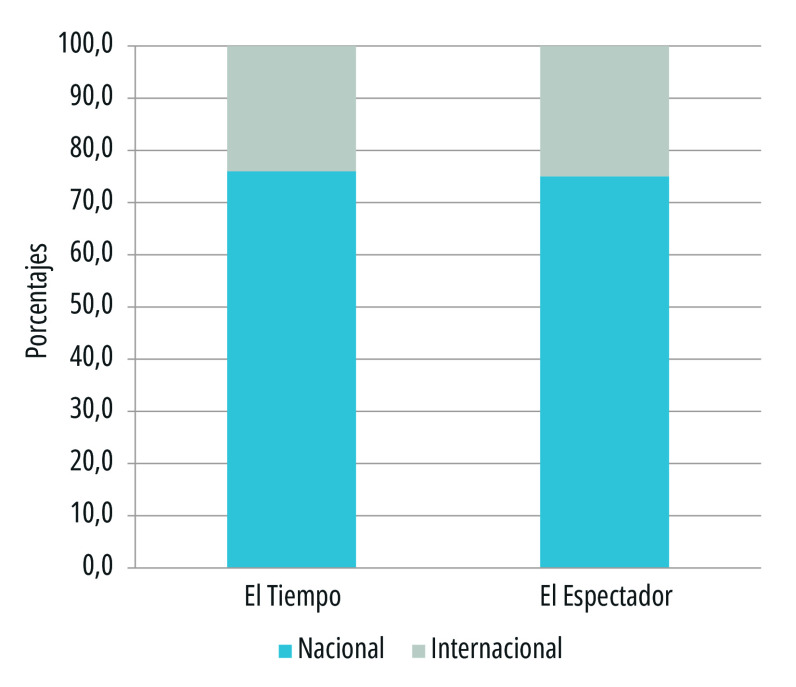
Tipos de cobertura nacional e internacional en los titulares de primera plana de los de los periódicos El Tiempo y El Espectador. Colombia, 15 de marzo a 15 de julio de 2020

En ese sentido, las agendas de este marco fueron muy similares en ambos periódicos. El 76% de los registros de *El Tiempo* hizo referencia a noticias originadas en el ámbito nacional, mientras que, en *El Espectador*, fue del 75%. En cuanto a la procedencia de informaciones desde el ámbito internacional, *El Tiempo* registró el 24% y *El Espectador*, el 25% (Figura 5). Estas coberturas citaban fuentes oficiales, especialmente, la OMS como actor preponderante.

Fuente: Elaboración propia

En el campo global, las informaciones provinieron con mayor frecuencia de estados como Rusia, EEUU y China. En el campo regional (latinoamericano), las informaciones relacionadas con Venezuela ocuparon un lugar importante en la agenda, teniendo en cuenta que Colombia ha sido la principal receptora de migrantes provenientes de ese país. Esto deja ver que los dos medios privilegiaron, en este segundo caso, el valor de noticiabilidad relacionado con la proximidad geográfica, no solo porque Venezuela comparta frontera con el país, sino porque los sujetos involucrados en la noticia hacen parte del ámbito territorial de difusión del medio, lo cual plantea una interacción entre los valores de proximidad y de relevancia, como lo señalan Túnez y Guevara^36^. En otras palabras, esto deja ver que la migración fue observada por los medios como un factor relevante en el encuadre de la información.

En los estudios sobre *framing* se ha determinado que los *frames* o encuadres no solo están plasmados en los textos. De hecho, en palabras de Teresa Sádaba Garraza^37^, los encuadres emergen en cuatro lugares en los procesos de información periodística: los periodistas, los textos, los receptores y la cultura. En esta investigación se ha puesto el foco en los textos, pero de alguna manera el análisis suele remitir a aspectos de la cultura, por ejemplo, a fenómenos en apariencia elementales como la forma en que los medios presentan gráficamente las informaciones para ser leídas por las audiencias.

**Figura 6:**
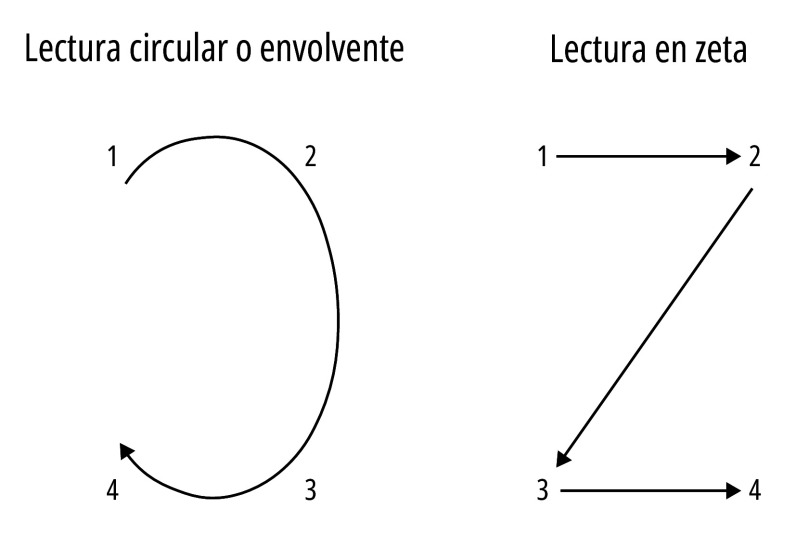
Recorrido visual de lectura.

En este orden de ideas, se incluyó el análisis de la ubicación de los titulares en la primera plana, por considerar que va en proporción directa al grado de importancia que el medio le quiere otorgar a la información. Esta ubicación depende de factores culturales: se suele recorrer (visualmente) los objetos de izquierda a derecha (cuando se lee, por ejemplo) y de arriba hacia abajo. De esta manera, el recorrido visual en la página de un periódico se plantea en dos maneras de lectura, como se señala en la Figura 6.

Fuente: Elaboración propia a partir de Leticia Medina Chávez^38^

**Figura 7:**
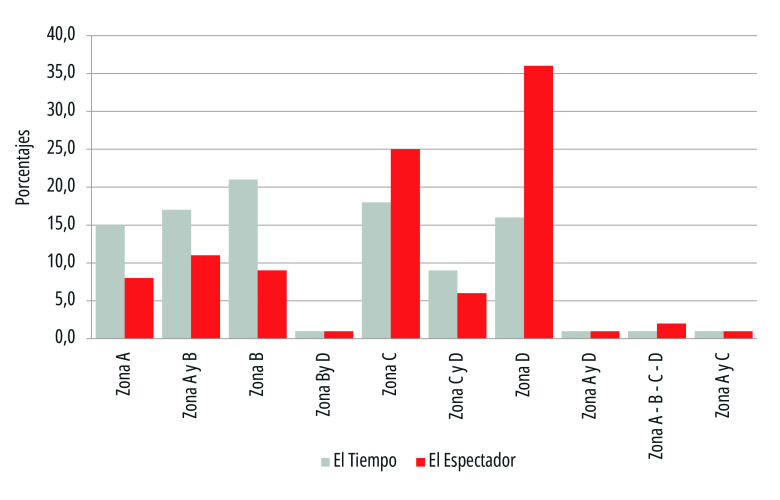
Ubicación de los titulares de primera plana de los periódicos El Tiempo y El Espectador. Colombia, 15 de marzo a 15 de julio de 2020.

A partir de estas consideraciones, en la página se parametrizaron cuatro zonas de acuerdo con su grado de visibilidad, así: Zona A, media alta visibilidad, que comprende el cuadrante derecho superior; Zona B, alta visibilidad, que comprende el cuadrante superior izquierdo; Zona C, baja visibilidad, que comprende el cuadrante inferior derecho, y Zona D, media baja visibilidad, que comprender el cuadrante inferior izquierdo. En esta revisión se registraron diez tipos de ubicación de los titulares, como se observa en la Figura 7.

Fuente: Elaboración propia.

En el diario *El Espectador*, cuyo formato es extra-tabloide, los titulares de prensa dedicados a la pandemia tuvieron preferentemente una ubicación en las zonas D (36%) y C (25%) de mediana y baja visibilidad, respectivamente, lo que denota que no siempre los titulares de este orden tenían toda la atención, pues también se destacaban temas del contexto colombiano al margen de la pandemia. En contraste, en *El Tiempo*, el 22% de los titulares sobre la emergencia sanitaria ocupó la zona B de alta visibilidad, en tanto que el 19% aparece en la zona C de baja visibilidad. Se observó que las variaciones en este aspecto guardaban relación directa con los casos de contagio.

Otro aspecto del análisis hace referencia a los términos más frecuentemente utilizados en los titulares, lo cual permitió observar a qué aspectos de la emergencia se hizo mayor alusión, es decir, a la manera cómo los medios construyeron representaciones del fenómeno. Por medio de las palabras, las personas no solo atribuyen denominaciones de los objetos, sino que los clasifican para lograr una mayor comprensión de la realidad. Nombres y palabra, como lo ha indicado Emanuela Pece^39^, representan categorías sociales mediante las cuales la gente organiza los objetos que componen su realidad en la vida cotidiana.

**Figura 8:**
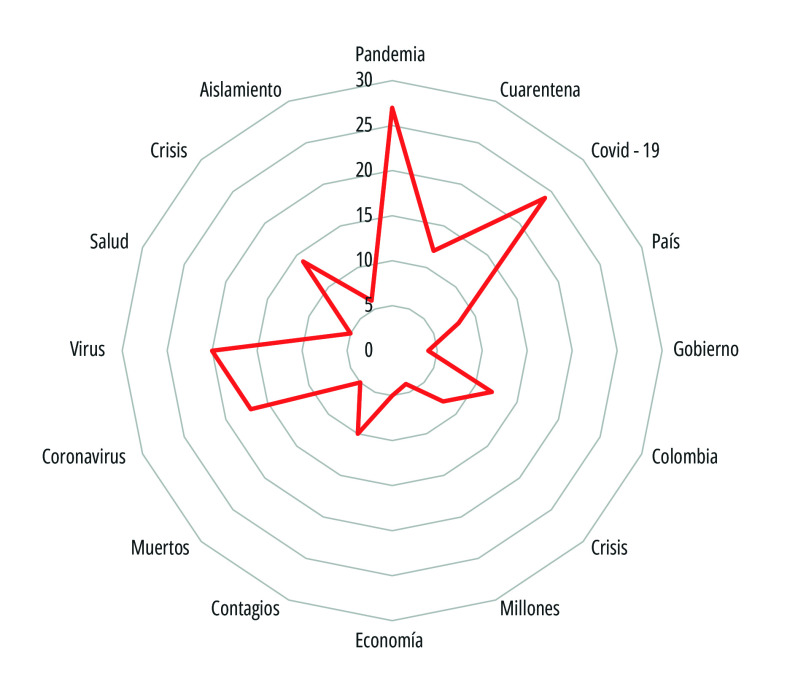
Palabras que con mayor frecuencia se utilizaron en los titulares de primera plana de los periódicos El Tiempo y El Espectador. Colombia, 15 de marzo a 15 de julio de 2020.

La terminología que con mayor frecuencia apareció en los titulares de primera plana durante en el periodo de marzo a junio de 2020 fueron pandemia, covid-19 y virus, evidentemente conceptos que directamente involucraban el tema central que afectaba mundialmente todas las esferas a nivel político, social y económico, como se ilustra en la Figura 8.

Fuente: Elaboración propia

Se observó que, de acuerdo con los titulares de la prensa analizados, el tono, el tipo del lenguaje, la cobertura y las fuentes de información de los periódicos de mayor circulación en Colombia permitieron identificar los principales criterios de encuadramiento, que se hicieron especialmente desde la incertidumbre, el conflicto y la seguridad.

Los resultados obtenidos dejan ver que, en efecto, los medios tienen la capacidad no solo de fijar la agenda de temas, sino de presentar en ella sus particulares formas de ver e interpretar la realidad, construyendo trazas de significados de acuerdo con los criterios que aplican a la hora de seleccionar las noticias que divulgarán en sus plataformas.

En el caso de *El Espectador* y *El Tiempo*, ha sido notorio identificar particulares *frames*, pese a que se trató de la cobertura sobre un acontecimiento común, cuyas informaciones provenían de fuentes similares, ligadas especialmente a organismos internacionales y a gobiernos locales. Los tonos informativos y apelativos en los titulares dejan ver que los medios buscan diferentes formas de influir en sus públicos y, en el caso de la pandemia, tanto para despertar la angustia y la incertidumbre, como para generar sentimientos de esperanza y superación de la crisis.

Puede considerarse que en los medios reposa gran parte de la responsabilidad frente a la manera como la gente enfrentó la crisis sanitaria, incluso, a sabiendas de que la agenda mediática suele anticiparse a la agenda del gobierno, que es el que tomas las medidas de contención, hayan sido estas acertadas o no.

## CONCLUSIONES

El análisis longitudinal permitió caracterizar el encuadre mediático de los titulares primera plana de los periódicos *El Tiempo* y *El Espectador* de Colombia, en relación con la crisis sanitaria del covid-19. Se evidenció la alta relevancia que se le otorgó a la pandemia en la definición de las agendas, lo cual se hizo evidente en el grueso número de registros localizados en las páginas de portada.

El tono negativo o pesimista primó en los titulares de los principales diarios escritos en Colombia, lo que se constituyó en una tendencia para visibilizar la crisis y el impacto como perjudicial en términos económicos, sociales y culturales. Esto se observó sobre todo cuando inició la emergencia sanitaria, pues luego de un periodo de desarrollo, el enfoque dejó de ser catastrófico y viró a lo esperanzador. Por ejemplo, en los picos de la pandemia, los titulares se tornaban alarmistas, pero cuando el panorama social cambiaba, se flexibilizaban las medidas de confinamiento y se apostaba a la reactivación económica, el tono tendía a ser positivo. Esto permitió observar una correlación entre la agenda de los medios y la agenda política.

En cuanto a los temas de encuadramiento, los diarios tuvieron enfoques distintos. En *El Espectador* primó la mirada desde lo social (31%) y lo sanitario (29%), mientras que en *El Tiempo* prevalecieron los temas sobre lo económico y social (25%). En relación con la tendencia del enfoque, *El Espectador* presentó una mirada más esperanzadora, que daba voz a las necesidades de las personas en situaciones más vulnerables de Colombia, como las comunidades más frágiles e impactadas por la pandemia. En contraste, *El Tiempo* asoció un tono de crisis, especialmente desde la perspectiva económica afectada hondamente por la aparición del covid-19.

Puede afirmarse que la cobertura de la pandemia, de acuerdo con el análisis del tono, tuvo tres enfoques: primero, una fase de tragedia; luego, una fase de incertidumbre y, finalmente, una fase de esperanza. Es decir, que los titulares fueron empleados durante las dos primeras fases como elementos expresivos, en tanto en la última, como elementos apelativos.

Las fuentes que prevalecieron en *El Tiempo* fueron las documentales (85%) por encima de las testimoniales (15%). Se evidenció una tendencia al cubrimiento desde la fuente oficial, sobre todo porque en un porcentaje considerable de titulares se destacaban las medidas del gobierno. En *El Espectador*, las fuentes documentales (57%) estuvieron casi a la par de las testimoniales (43%). Este medio destacó, más que *El Tiempo*, la voz de la ciudadanía, de la comunidad científica, de quienes trabajan en el campo de la salud o de forma independiente, de la clase política y de la diversidad de fuentes humanas que narraban la realidad desde el confinamiento.

En resumen, en cuanto al manejo de fuentes de información, el enfoque marcó grandes diferencias, pues *El Tiempo* privilegió el relato desde la institucionalidad, en tanto que *El Espectador* lo hizo desde la colectividad. Ahora bien, a la luz del pregonado equilibrio informativo que se demanda de los medios de prensa, puede afirmarse que *El Espectador* atendió de mejor manera a este principio.

En relación con la ubicación de los titulares, en el diario *El Tiempo* prevalecieron las zonas de alta visibilidad, mientras que en *El Espectador* fue más notable la zona D, de media baja visibilidad, y se distinguió porque su enfoque central no privilegió siempre la emergencia sanitaria, sino a otras realidades del acontecer nacional. Puede esto interpretarse como la intención de ampliar los enfoques informativos hacia otros tópicos distintos del asunto más relevante en términos de que, a pesar de la pandemia, la vida social seguía adelante.

En suma, se observó un encuadramiento de la pandemia distinto en ambos medios, con marcadas diferencias de tono, lenguaje, cobertura, abordaje temático y visibilidad del titular. Sin embargo, a pesar de sus diferencias, los encuadres coincidieron en algunas etapas, especialmente frente a la incertidumbre y la crisis. Aunque cada medio intentó vincular miradas esperanzadoras, no se alejaron del tono negativo y apesadumbrado que, en medio de la pandemia, se suscitó con la sobreinformación. Es importante destacar que, en medio de este panorama, los medios construyeron una narrativa soportada en fuentes confiables y de reconocimiento social.
